# Effects of Boron on the Emergence and Allocation of Metabolic Compounds in Two Herbaceous Cotton Cultivars

**DOI:** 10.3390/plants14040576

**Published:** 2025-02-13

**Authors:** Roberta Possas de Souza, Maycon Anderson de Araujo, Lucas Baltazar Longhi, Isabella Fiorini de Carvalho, Bruno Bonadio Cozin, Liliane Santos de Camargos

**Affiliations:** Department of Biology and Zootechny, School of Engineering, São Paulo State University (UNESP), Ilha Solteira 15385-000, Brazil; roberta.possas@unesp.br (R.P.d.S.); maycon.araujo@unesp.br (M.A.d.A.); lucas.longhi@unesp.br (L.B.L.); if.carvalho@unesp.br (I.F.d.C.); bruno.bonadio-cozin@unesp.br (B.B.C.)

**Keywords:** soil contamination, micronutrient toxicity, plant physiology, N metabolism

## Abstract

High boron (B) concentrations in the soil can cause toxic effects to plants, so herbaceous cotton (*Gossypium hirsutum* latifolium Hucth) is a crop sensitive to such stress. Thus, this study aimed to evaluate the emergence, partitioning, and allocation of metabolic compounds of two herbaceous cotton cultivars subjected to B treatments. The experiment was carried out in a greenhouse, under a completely randomized design in a 2 × 4 factorial scheme, using two cultivars and four concentrations of B in the form of boric acid: 0.5 (control), 30, 60, and 120 mg dm^−3^ B. The increase in the concentration of B in the soil generated a significant toxic effect on the growth and biomass of the cotton plant. Cultivar 1—TMG 50 WS3 obtained greater emergence and shoot growth, while cultivar 2—FM 911 GLTP invested in roots; however, in both cultivars, B remained accumulated in the shoot. There was an increase in amino acids in the roots and a decrease in proteins and phenolic compounds in leaves and cotyledons. It was concluded that the seedlings presented satisfactory emergence up to 60 mg dm^−3^ B, and that among the cultivars there are distinct responses to B application.

## 1. Introduction

Boron (B) is a semi-metal widely used in the agricultural industry and is an important micronutrient for plant growth and development [1]. It has functions related to the cell wall structuring and participation in the metabolism of nucleic acids, sugars, phenolic compounds, protein synthesis, and hormonal regulation [2]. Furthermore, tropical sandy soils are often deficient in this element, which is a problem affecting the yield of crops such as cotton [3]. In this context, adequate concentrations of B can increase the synthesis of auxins, gibberellins, and cytokinins in plants, thus increasing crop productivity [4]. However, the threshold between B deficiency and toxicity is very narrow [5], so that higher concentrations in the soil can lead to the appearance of toxic effects, damaging various vegetative and reproductive aspects of plants [2], due to its binding with multiple hydroxyl groups in the cis configuration, generating such known effects [6].

Cotton (*Gossypium hirsutum latifolium* Hucth) is one of the best-known fibers in the world, and it is suggested that its domestication began 4000 years ago in Mesoamerica [7], presenting different growth cycles and cultivars, a wide variety of genotypes, and many possibilities for use in industry, agriculture, and commerce [8]. Furthermore, cotton is a crop that is sensitive to biotic and abiotic stresses, which is a problem that affects its yield in different countries, generating opportunities for research addressing the crop’s yield potential, involving different branches of agricultural science, such as crop physiology [9]. Furthermore, B application in desiccation for the management of phytopathogens is a common practice in agriculture [10], highlighting the importance of studies that observe the emergence of seeds under the element’s influence.

Among the different cultivars available, the TMG 50 WS3 cultivar stands out, containing WideStrike^®^ 3 technology that guarantees protection against lepidopteran insects through the expression of the insecticidal proteins Cry1Ac, Cry1F, and Vip3A19, derived from *Bacillus thuringiensis* [11]. This technology ensures that the cotton plant remains healthy and less susceptible to environmental stresses during its growth, reaching greater production potential [11]. Another notable cultivar is FM 911 GLTP, which has the GlyTol^®^, LibertyLink^®^, GlyTol LibertyLink^®^ and GlyTol LibertyLink Twinlink Plus^®^ technologies, which ensure selectivity to glyphosate-based herbicides and the Liberty^®^ herbicide, allowing rotation of both, and providing protection against caterpillars using Cry1Ab and Cry2Ae proteins with the *Vip3A* gene [12]. Therefore, due to their high use in agriculture and greater productivity, both cultivars were selected for the present study.

Thus, the application of emergency tests that can compare different cultivars is interesting, even from a commercial point of view, since they check the proportion of live seeds in a given batch capable of producing normal plants, leading to greater agricultural productivity and lower costs related to production [13]. However, such tests are usually performed under optimal conditions, which is a limitation, since this context cannot be represented in the field [14]. Therefore, tests carried out under adverse conditions—such as micronutrient toxicity—help simulate less favorable environmental conditions [13], in addition to being an effective and economical bioassay to evaluate the potential toxicity of a compound [15], such as different concentrations of B.

Therefore, the hypothesis developed for this study is that increasing soil B concentration can affect the metabolism and emergence rate of two upland cotton cultivars in different ways. The purpose of this study was to evaluate the emergence, partitioning, and allocation parameters of nitrogen compounds in two herbaceous cotton cultivars subjected to treatments with B in the soil.

## 2. Results

**Figure 1:**
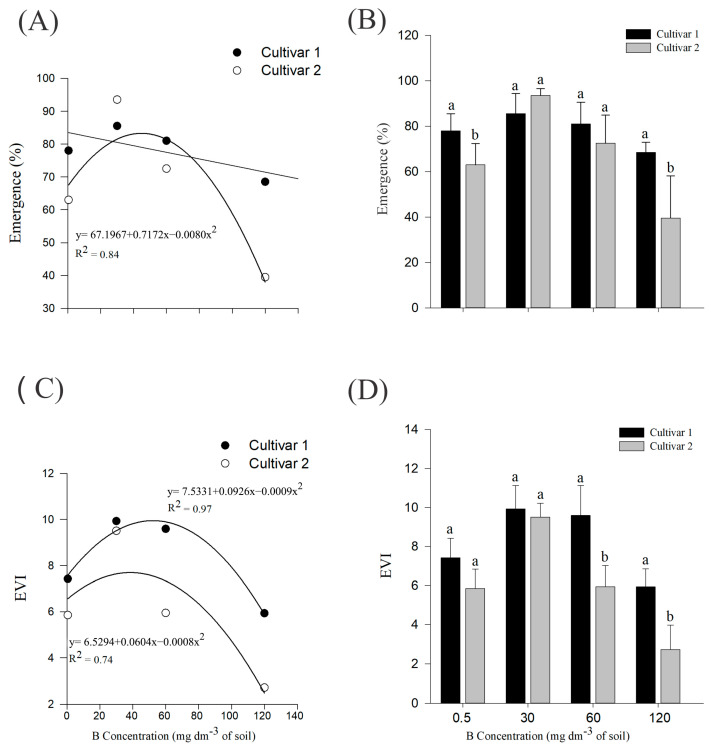
(**A**,**B**) Emergence percentage; (**C**,**D**) emergence velocity index of *G. hirsutum* seedlings subjected to B treatments. Means represented by different letters differ from each other in each treatment by the Tukey test at 5% probability (*p* ≤ 0.05). A plot without a regression equation represents data that did not fit the linear or quadratic curve.

Regarding the emergence percentage (Figure 1A), cultivar 2 reached a maximum point at 44.83 mg dm^−3^ B. Both cultivars differed significantly in the control and 120 mg dm^−3^ B treatments (Figure 1B).

There was a statistical difference for the first and second cultivars in relation to the emergence velocity index—EVI (Figure 1C), presenting, respectively, maximum points at 51.44 and 37.75 mg dm^−3^ B. Both cultivars differed significantly in the 60 and 120 mg dm^−3^ B treatments (Figure 1D).

**Figure 2:**
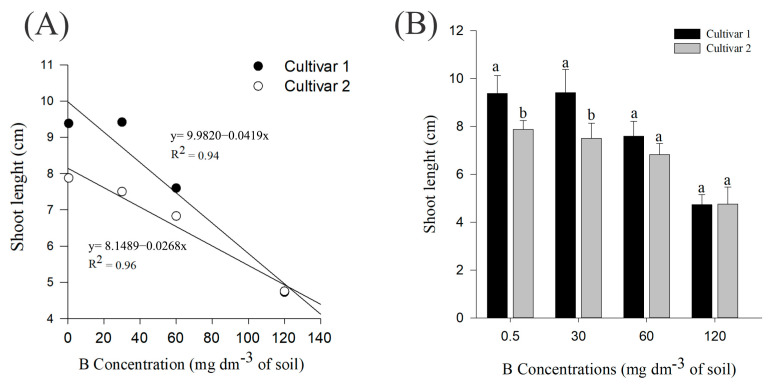
(**A**,**B**) Shoot length of *G. hirsutum* seedlings subjected to treatments of B. Means represented by different letters differ from each other in each treatment by the Tukey test at 5% probability (*p* ≤ 0.05).

**Figure 3:**
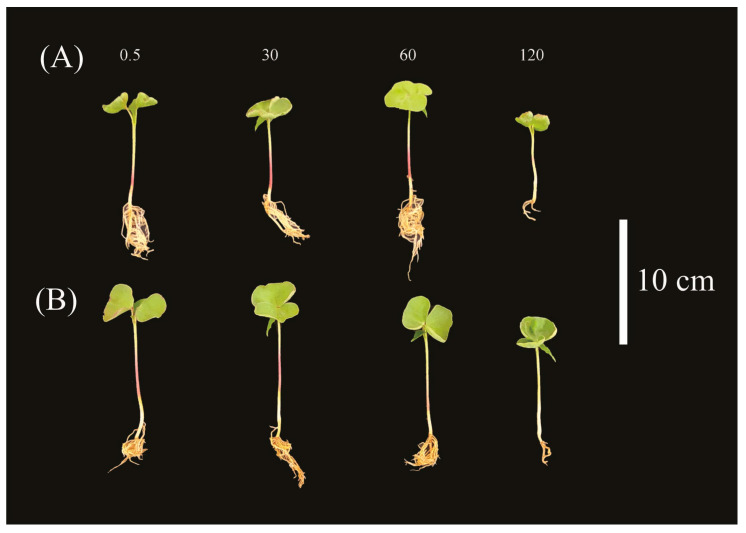
(**A**) *G. hirsutum* seedlings of cultivar 1 and (**B**) cultivar 2 throughout the four B treatments.

While B concentrations increased, there was a decrease in the shoot length in both cultivars (Figure 2A), and a decrease in seedling size could be visibly observed (Figure 3A,B). There was a statistical difference between both cultivars in the control and 30 mg dm^−3^ B treatments (Figure 2B), so that cultivar 1 presented higher averages in relation to the second cultivar, in all variables. For the roots, the interaction between treatments and cultivars was not significant, so both factors were analyzed individually. When cultivars were presented as one, there was a decline in the root system throughout treatments (Figure S1A—Supplementary Materials), so that cultivar 2 presented higher averages in relation to cultivar 1 (Figure S1B—Supplementary Materials), regardless of the treatments. Furthermore, there was no significant interaction between treatments and cultivars for shoot dry mass; however, there was a decline in biomass (Figure S1C—Supplementary Materials), regardless of cultivars. For the root (Figure S1E,F—Supplementary Materials), there were no significant results for any of the studied factors (cultivars and treatments), as well as for the interaction between them.

Regarding the quantification of B in cotton tissues, we noted that for both cultivars, the application of treatments resulted in a predominant increase in the micronutrient for the shoot and a decrease in the root system (Table 1). In the shoot, cultivars 1 and 2 presented values five and four times higher in relation to the control, respectively, in the treatment with 120 mg dm^−3^ B.

### 2.1. Physiological Analysis: Quantification of Amino Acids

**Figure 4:**
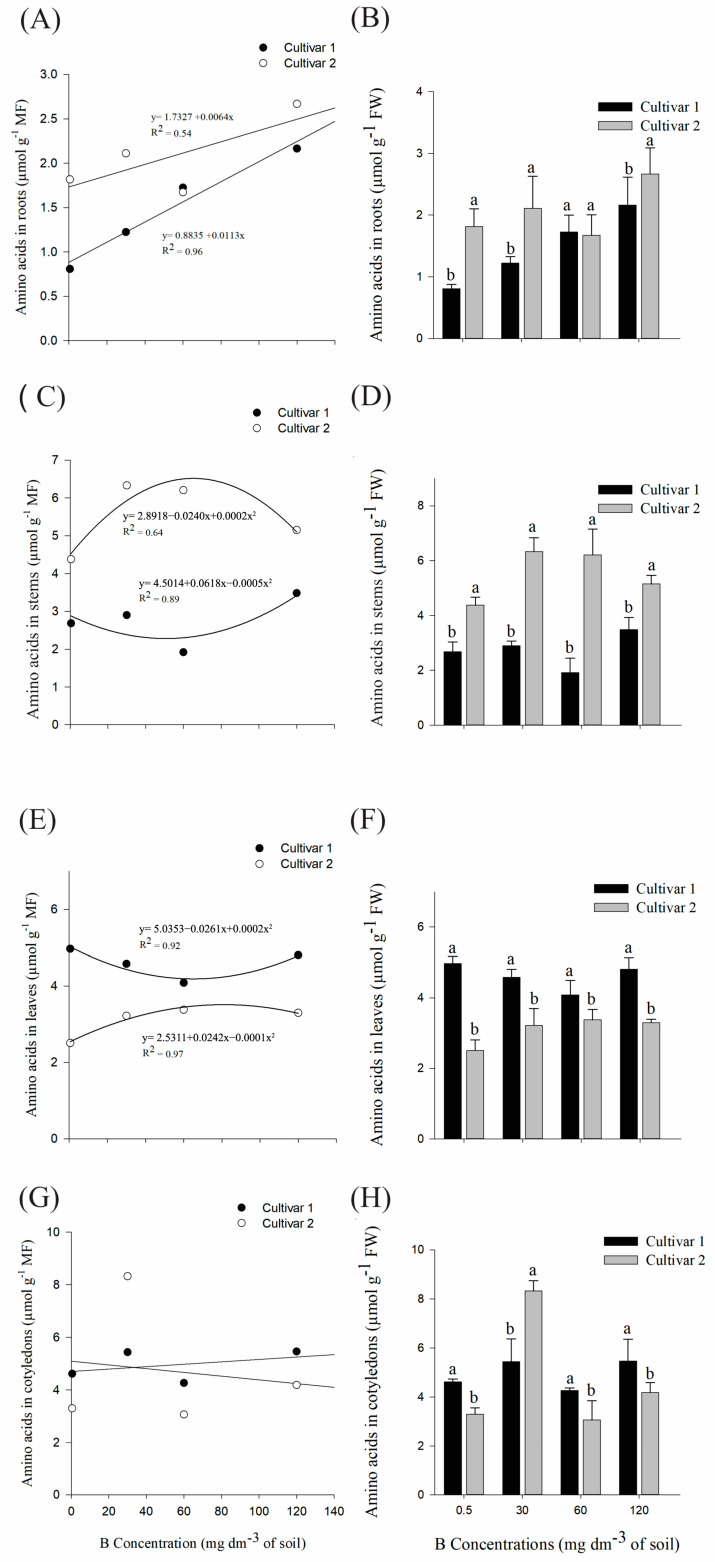
(**A**,**B**) Quantification of amino acids in roots; (**C**,**D**) stems; (**E**,**F**) leaves and (**G**,**H**) cotyledons of *G. hirsutum* seedlings subjected to high concentrations of B. Means represented by different letters differ from each other in each treatment by the Tukey test at 5% probability (*p* ≤ 0.05). Plots without a regression equation represent data that did not fit the linear or quadratic curve.

Throughout the treatments, there was an increase in amino acids in the roots of both cultivars (Figure 4A). In addition, when compared in each treatment, the averages of the cultivars differed statistically in the control treatments, 30 and 120 mg dm^−3^ B (Figure 4B). In the stems, cultivar 1 reached a minimum point at 61.8 mg dm^−3^ B, while cultivar 2 reached a maximum point at 60 mg dm^−3^ B (Figure 4C). There was a statistical difference in all treatments (Figure 4D), so cultivar 2 presented the highest averages in both addressed organs.

Cultivar 1 reached a minimum point of 65.25 mg dm^−3^ B in the leaf system (Figure 4E), and the second cultivar showed a maximum point of 121 mg dm^−3^ B. When comparing the averages of both cultivars, cultivar 1 presented higher averages in all treatments in relation to the second cultivar (Figure 4F). For cotyledons, except for the 30 mg dm^−3^ B treatment, cultivar 1 also showed higher averages compared to cultivar 2 (Figure 4H).

#### 2.1.1. Physiological Analysis: Quantification of Total Soluble Proteins

**Figure 5:**
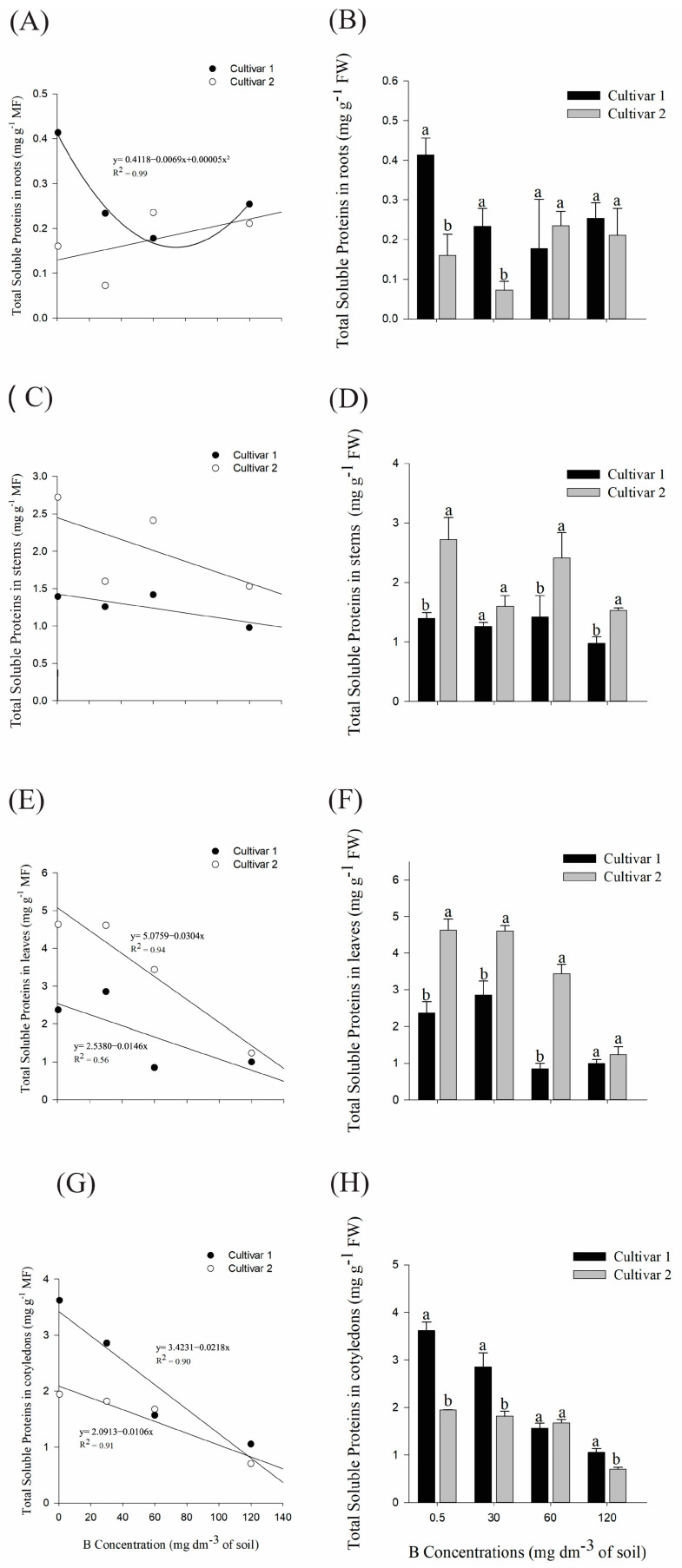
(**A**,**B**) Quantification of total soluble proteins in roots, (**C**,**D**) stems, (**E**,**F**) leaves, and (**G**,**H**) cotyledons of *G. hirsutum* subjected to high concentrations of B. Means represented by different letters differ from each other in each treatment by the Tukey test at 5% probability (*p* ≤ 0.05). Plots without a regression equation represent data that did not fit the linear or quadratic curve.

Cultivar 1 showed a minimum point of 69 mg dm^−3^ B in the roots (Figure 5A), and presented higher averages in relation to cultivar 2, in the control and 30 mg dm^−3^ B treatments (Figure 5B).

In the stems, cultivar 2 presented higher protein averages compared to cultivar 1 in the control, 60, and 120 mg dm^−3^ B treatments (Figure 5D). Except for the highest dose of B, this pattern was also observed for the leaves (Figure 5F); in addition, the increase in B in the soil also led to a decrease in proteins in the leaf system (Figure 5E) and cotyledons (Figure 5G) for both cultivars. For cotyledons, the cultivar averages differed statistically in the control treatment and in 30 mg dm^−3^ B (Figure 5H), so that cultivar 1 presented higher means.

#### 2.1.2. Physiological Analysis: Quantification of Phenolic Compounds

**Figure 6:**
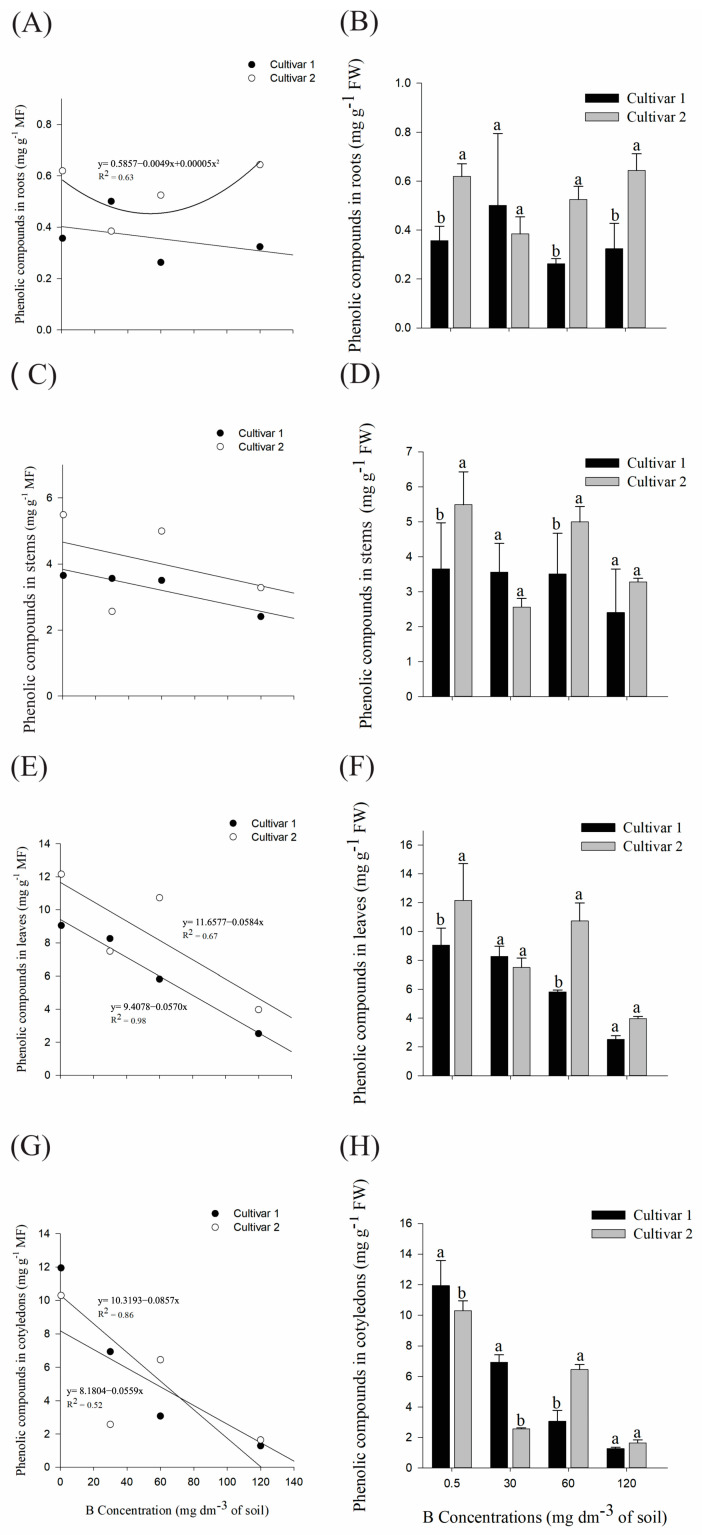
(**A**,**B**) Quantification of phenolic compounds in roots, (**C**,**D**) stems, (**E**,**F**) leaves, and (**G**,**H**) cotyledons of *G. hirsutum* subjected to high concentrations of B. Means represented by different letters differ from each other in each treatment by the Tukey test at 5% probability (*p* ≤ 0.05). Plots without a regression equation represent data that did not fit the linear or quadratic curve.

In the roots, cultivar 2 showed a minimum point at 49 mg dm^−3^ B (Figure 6A) and when compared in each treatment, both cultivars differed in the control, 60, and 120 mg dm^−3^ B treatments (Figure 6B). In the stem and leaf system (Figure 6D,F), the second cultivar presented higher averages in relation to cultivar 1 in the control and at 60 mg dm^−3^ B treatments. Furthermore, there was a decrease in phenolic compounds in the leaves (Figure 6E) and cotyledons (Figure 6G) of both cultivars as the B dose applied to the soil increased.

The averages of both cultivars differed in the first three treatments in the cotyledons (Figure 6H), so that, except for the 60 mg dm^−3^ B treatment, cultivar 1 presented higher means in relation to the second.

### 2.2. Pearson Correlation and Principal Component Analysis (PCA)

**Figure 7:**
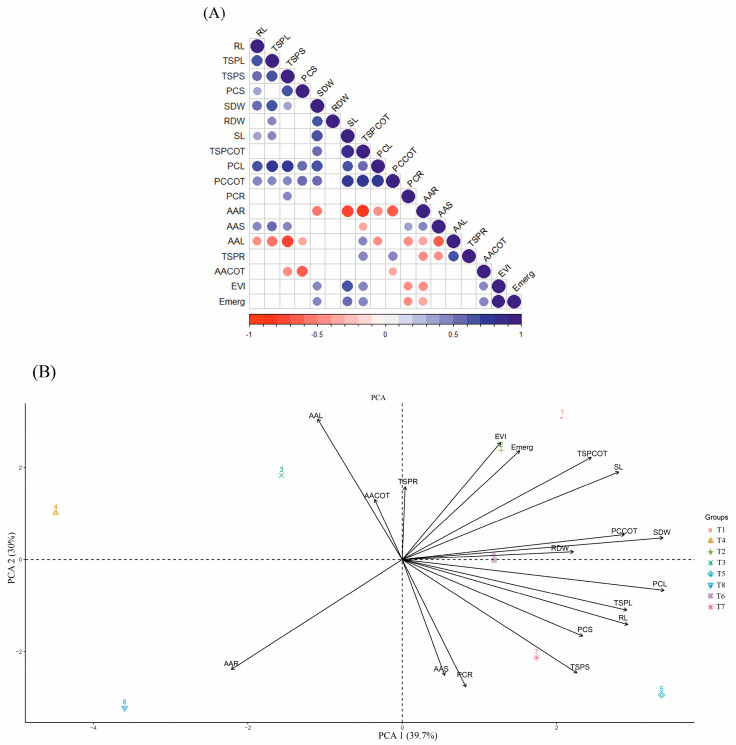
(**A**) Correlation matrix; blank spaces indicate non-significant correlation by Pearson’s correlation (*p* < 0.05); (**B**) Principal Component Analysis. **RL**—root length. **TSPL**—Total soluble proteins in leaves. **TSPS**—Total soluble proteins in stem. **PCS**—Phenolic compounds in stem. **SDW**—shoot dry weight. **RDW**—root dry weight. **SL**—shoot length. **TSPCOT**—Total soluble proteins in cotyledons. **PCL**—Phenolic compounds in leaves. **PCCOT**—Phenolic compounds in cotyledons. **PCR**—Phenolic compounds in roots. **AAR**—Amino acids in roots. **AAS**—Amino acids in stems. **AAL**—Amino acids in leaves. **TSPR**—Total soluble proteins in roots. **AACOT**—Amino acids in cotyledons. **EVI**—Emergence velocity index. **Emerg**—Emergence. **T1**—Cultivar 1—0.5 mg dm^−3^ B. **T2**—Cultivar 1—30 mg dm^−3^ B. **T3**—Cultivar 1—60 mg dm^−3^ B. **T4**—Cultivar 1—120 mg dm^−3^ B. **T5**—Cultivar 2—0.5 mg dm^−3^ B. **T6**—Cultivate 2—30 mg dm^−3^ B. **T7**—Cultivar 2—60 mg dm^−3^ B. **T8**—Cultivar 2—120 mg dm^−3^ B.

The concentrations of phenolic compounds in cotyledons and leaves showed a positive correlation (Figure 7A) with each other and with many variables such as total soluble proteins in cotyledons, leaves and stem; shoot length and dry mass; root length; and phenolic compounds of the stem. It was observed that amino acids in leaves were the variable that presented the most negative correlations, relating to amino acids in the stem and roots, phenolic compounds in roots, leaves and stem, and a strong significant correlation with total soluble proteins in the stem and leaves, and root length. A strong negative correlation was also observed between the concentrations of amino acids in the roots and the concentrations of phenolic compounds in leaves and cotyledons, total soluble proteins in cotyledons, and shoot dry weight and length (Figure 7A).

The first two principal components (Figure 7B) explain 69.7% of the data variation. The cultivar 1 treatments at concentrations of 0.5 and 30 mg dm^−3^ B were those that most influenced the variation of data in relation to emergence and EVI, while the treatments with cultivar 2 at concentrations of 0.5 and 60 mg dm^−3^ B significantly influenced the variation of total soluble proteins and phenolic compounds in leaves and stems, and root length. The maximum concentration of cultivar 1 did not influence any variable, while the concentration of amino acids in the roots was mainly influenced by the application of 120 mg dm^−3^ B in cultivar 2.

## 3. Discussion

B is an element capable of improving seedling growth, productivity, and biofortification [16]; however, concentrations above 2.0 mg L^−1^ can already reduce crop productivity, so the response to toxicity depends on several factors, such as species and cultivars, and plant growth and development parameters [17]. In seeds, the application of B can result in an improvement in emergence, growth, and even biomass production, as observed for wheat [16]. For safflower (*Carthamus tinctorius* L.) this is also valid, so stimulating concentrations (5–10 mg L^−1^) can improve the germination index and the average germination time of seedlings, in addition to reducing the negative effects of pathogens [18].

For both cotton cultivars studied, there was a peak in emergence parameters, so that above the concentrations determined by the maximum point, there was a general decline in seedling emergence. The inhibition or delay of germination at higher concentrations of B is a topic addressed in the literature, and this pattern is also observed for wheat and barley [16,19]. The plant general development also declined, as observed in Figure 2, Figure 3, and Figure S1—Supplementary Materials, which can be explained by the decrease in cell division and tissue expansion, a problem caused by the B toxicity [17,20]. Also, for rice seedlings [21] and for 8-week-old safflower [20], high concentrations of B led to reduced seedling growth.

Thus, the concentrations found from the maximum points in this work, despite initially stimulating cotton emergence, did not allow adequate seedling development, directly interfering with their growth and dry biomass. Both cultivars showed accumulation of B in the shoots; however, in general, cultivar 1 adopted as its main strategy the shoot development stimulation, while cultivar 2 invested in root elongation. Thus, the growth capacity of plants in soils with high concentrations of B varies between species or even cultivars within the same crop, and, for tolerant cultivars, the basis for tolerance is the B efflux capacity through transporters or reduction of B channels [2,22]. In addition, transcription factors or morphological changes may occur, enhancing the tolerance of certain species or cultivars under such conditions [2].

As it is a micronutrient, vegetables require B in small concentrations, and therefore, any imbalance between its availability and need can cause problems [2], so that the element’s binding to compounds that have two hydroxyl groups triggers changes in the cell wall structure and interruption in metabolism through binding to the ribose molecules of NADH, NADPH, and ATP, causing the known toxic effects [23]. However, when we address the physiological consequences related to excess B in plant tissues, related research mainly focuses on its involvement in the structure and function of cell walls, so that other interactions of B within cells are still a poorly understood topic [24].

The cultivars studied in the present research presented opposite responses regarding the distribution of amino acids in stems and leaves due to the effect of B. For plants, the demand and local concentration of amino acids occur in response to the availability of nitrogen and carbon, and its regulation occurs through the biosynthesis and degradation of amino acids and proteins, as well as through transport processes [25,26]. In seedlings, until the leaves are fully developed and functional, the energy demand is met by the oxidation of amino acids and other storage compounds [27]; thus, in photosynthetically active and growing cells, amino acid biosynthesis is regulated to provide substrates for protein synthesis [26]. For both cultivars, there was an increase in amino acids in the roots, so that, during stress, pools of all amino acids are induced [26]. Furthermore, these compounds have an antioxidant effect and are associated with the development of this organ in plants [28,29], which explains the greater root growth for cultivar 2, since it presented, in almost all treatments, greater quantification of amino acids in relation to cultivar 1.

When we approach the germination period, amino acids are provided through the degradation of storage proteins, and these are used for the biosynthesis of other proteins necessary for the plant development [26]. Under abnormal conditions that deprive the plant of its normal life cycle, proteins are degraded, and their amino acids’ oxidation produces the energy required to supply the needs of stressed organs [26]. The response observed in the present study, regarding the decrease in the content of total soluble proteins in leaves and cotyledons, with the increase in B in the soil, demonstrates the possible interference of the micronutrient in the cellular protein homeostasis, a very common response in the presence of environmental pollutants, such as heavy metals [30]. Furthermore, the conformation of a protein can be affected by a series of stress factors that compromise the folding process of newly synthesized and existing proteins [30,31], so that such proteins can aggregate and/or interact inappropriately with other cellular components, becoming cytotoxic [32].

Phenolic compounds are antioxidant products of secondary metabolism and have functions associated with defense against environmental aggressions [33]. B is an important element for phenolic metabolism in vascular plants, so that its deficiency induces the accumulation of phenolics by activating the enzyme phenylalanine ammonium lyase and increasing the polyphenol oxidase activity, an enzyme that catalyzes the oxidation of phenolic compounds [17,34]. However, under excess B conditions, the opposite effect may occur, leading to a decrease in both enzymatic activities, as observed for the roots of *Linum usitatissimum* [35]. In the present study, cotyledons and leaves present lower concentrations of phenolic compounds as B increases in the soil, which may be due to this effect. Furthermore, B accumulation in the plant shoot can lead to oxidative damage, causing osmotic imbalances [17] that can undermine the plant’s defense.

Furthermore, phenolic compounds demonstrated a great influence on the data of this work, due to their high number of positive correlations with other variables, such as total soluble proteins. It is known that the interactions of phenolic compounds and proteins affect the structure of proteins and the content of free polyphenols, as well as their antioxidant capacity and bioavailability, in the case of foods [36]. In addition, high concentrations of phenolic compounds are associated with the defense functions of plants, generating thick and dense leaves with low growth [37], which can also explain the positive correlation between phenolic compounds in leaves and cotyledons and shoot dry weight, reinforcing the importance of these compounds to promote the balance between growth and defense functions, especially under stressful environmental conditions.

The B concentrations used in this study led to an imbalance in physiological parameters and changes in seedling growth. From this study, it is possible to understand that the treatment of 30 mg dm^−3^ B is already capable of harming herbaceous cotton seedlings, compromising the synthesis of proteins, amino acids, and phenolic compounds. These data can be corroborated by anatomical studies, which demonstrate that such concentration can cause several changes in plants, such as the decrease in the thickness of the abaxial epidermis in leaves and the decrease in primary xylem in roots, as observed for *Calopogonium mucunoides* [38]. For cotton, the treatment of 60 mg dm^−3^ B can be seen as the limit to be supported, since, despite affecting N metabolism, the seedlings presented satisfactory performance for emergence parameters. Doses of 120 mg dm^−3^ B were shown to affect the general development of seedlings, which was also observed for *Crotalaria juncea*, followed by an increase in B concentration in the shoot and a negative effect on N metabolism [39], which is also observed in this work.

## 4. Materials and Methods

### 4.1. Soil Preparation

The experiment was conducted in a completely randomized design in a greenhouse in Ilha Solteira—SP, Brazil (20°25′58″ S, 51°20′33″ W) covered with 1000-micron plastic film and automatic irrigation three times a day for 10 min, maintaining the temperature under 30 °C. This work presents a 2 × 4 factorial scheme, with 2 cultivars of herbaceous cotton: TMG 50 WS3 (Cultivar 1) and FM 911 GLTP (Cultivar 2), and 4 concentrations of B in the form of boric acid applied to the soil (H_3_BO_3_—61.83 g mol^−1^), being 0.5 (control containing the minimum amount of B to avoid deficiency [40]), 30, 60, and 120 mg dm^−3^ B.

The soil used was classified as Oxisol [41] and was collected from the experimental area of the teaching, research and extension farm, vegetable production sector, Selvíria, MS, Brazil. The granulometric analysis of the soil [42] verified the following proportions: 121, 876 and 3 g kg^−1^ of clay, sand, and silt, respectively. The chemical attributes of the soil showed the following values [43]: pH = 5.5 (determined with CaCl_2_ 0.01 M); organic matter = 15.0 g dm^−3^; P = 6.0 mg dm^−3^ (resin); K = 1.0, Ca = 8 e Mg = 9, mmol_c_ dm^−3^ (resin); B = 0.04 mg dm^−3^ (warm water); Cu = 0.4, Fe = 9, Mn = 3.4 e Zn = 1.2 mg dm^−3^ (DTPA); potential acidity 13.0 mmol_c_ dm^−3^ (SMP buffer); Al = 0.0 mmol_c_ dm^−3^; the sum of bases 13.1 mmol_c_ dm^−3^; cation exchange capacity 26.1 mmol_c_ dm^−3^ and base saturation 50%.

The soil was sieved, homogenized, and subjected to the application of B doses so that, after the application of the treatments, it remained for 15 days in plastic bags in a period of micronutrient stabilization [44]. The available B levels in the soil after stabilization were 0.24, 11.97, 12.08, and 12.1 mg dm^−3^ B for treatments 0.5, 30, 60, and 120 mg dm^−3^ B, respectively; these concentrations represent 0.48, 23.94, 24.16, and 24.2 kg ha^−1^ B.

The soil was transferred to propylene seedling boxes with a capacity of 200 cells (each cell containing a diameter of 2.3 cm, 4 cm in depth, and 13 mL in volume). The seeds were deposited directly in the soil in individual cells, and each germination tray consisted of one treatment, being divided into 4 replicates containing 50 cells each, forming an experimental unit (32 experimental units in total).

### 4.2. Emergence Evaluations

At the end of 15 days after sowing, the emergence velocity index (EVI), emergence percentage (%), shoot dry weight (g), root dry weight (g), shoot length (cm), and root length (cm) were evaluated.

The seedlings considered to be emerged were those with cotyledons exposed above 1 cm from the soil [45,46]. The EVI was calculated using Equation (1) described by Maguire (1962) [47]:

EVI = (E1/N1 + E2/N2 + … + E15/N15)(1)

EVI = emergence velocity index;

E = number of emerged plants;

N = day of counting after sowing.

The percentage of emergence was calculated following Equation (2):

EP = Total number of seedlings emerged/Total number of seeds planted × 100(2)

Length assessments were performed with the aid of a ruler [45], and part of the collected material was taken to forced circulation ovens at 60 °C for 72 h to obtain dry biomass. In addition, a nutritional analysis was performed to obtain B in the plant tissues.

### 4.3. Partitioning and Allocation of Metabolic Compounds

At the end of the experiment, part of the material was used for physiological analysis, being collected and partitioned into leaves, stem, cotyledons, and roots so that all structures were washed in running water and dried on paper towels. The fresh material was used for the extraction of soluble compounds and quantification of metabolic compounds.

### 4.4. Extraction of Soluble Compounds

The Bieleski and Turner method [48] was used to extract soluble compounds, so that for 1 g of fresh material, 10 mL of an MCW solution (60% methanol, 25% chloroform, and 15% H_2_O) was added. Thus, the material was macerated and centrifuged for 15 min at 10,000 rpm, and subsequently 1 mL of chloroform and 1.5 mL of H_2_O were added for each 4 mL of supernatant. After 48 h, phase separation occurred so that the water-soluble phase was used for the quantification of amino acids [49] and phenolic compounds [50]. To the precipitate, 10 mL of 0.1 M NaOH was added, and the tubes were homogenized and centrifuged (15 min at 10,000 rpm). The supernatant was used for protein quantification [51].

### 4.5. Quantification of Total Soluble Amino Acids

To the sample, 500 µL of 0.2 M citrate buffer at pH 5.0, 200 µL of 5% ninhydrin in methylglycol, and 1 mL of 0.0002 M KCN were added. The test tubes were placed in a water bath at 100 °C for 20 min and remained for 10 min at room temperature. Subsequently, 1 mL of 60% ethanol was added. Readings were performed in a spectrophotometer (λ = 570). The results were determined through the curve using Methionine and expressed in µmol g^−1^ FW (fresh weight) [49].

### 4.6. Quantification of Phenolic Compounds

To the sample, 1 mL of 0.2 N Folin Ciocalteu reagent was added, and after 4 min, 800 µL of a saturated sodium carbonate solution (75 g in 1 L of water) was added. After 2 h at room temperature, readings were taken on a spectrophotometer (λ = 760 nm), and the results were expressed in µmol g^−1^ MF after adjustment to the curve using gallic acid [50].

### 4.7. Quantification of Total Soluble Proteins

To the sample, 1 mL of Bradford reagent was added, and readings were performed in a spectrophotometer (λ = 595 nm) after 3 min at room temperature. The results were expressed in mg g^−1^ FW after being adjusted to the Albumin curve [51].

### 4.8. Statistical Analysis

The results were subjected to analysis of variance (ANOVA) using R 4.3.0 software (Package ExpDes.pt) and subsequently submitted to regression analysis to verify the adequacy of the B concentrations to the curve, and to a Tukey test (*p* < 0.05) to compare the cultivars within each treatment. The graphs were made using SigmaPlot 12.5 software. The data were also subjected to multivariate analysis using Pearson correlation (*p* < 0.05) and Principal Component Analysis (PCA). The graphs were made using R 4.3.2 software (Packages: corrplot, FactoMineR and factoextra).

## 5. Conclusions

Both cultivars showed toxic effects caused by B, which resulted in an accumulation of B in the shoots and a decrease in the roots. However, when compared, the cultivars developed different strategies to deal with the toxicity conditions, so cultivar 1 stands out in relation to shoot growth and emergence, while cultivar 2 presents greater investment in root growth. It is concluded that up to a dose of 60 mg dm^−3^ B in the soil, there was an acceleration of the speed and percentage of cotton emergence; however, the higher concentrations of B led to a decrease in length and dry biomass, in addition to the reduction of total soluble proteins and phenolic compounds in cotyledons and leaves of both cultivars, impairing the development of seedlings. The increase in amino acids in roots may be a survival strategy adopted by cotton; however, the cultivars presented a pattern of opposite results in stems and leaves, clarifying that B influences the cultivars in different ways.

## Figures and Tables

**Table 1:** B accumulation (mg B kg^−1^ DW) in cultivar 1 and cultivar 2 seedlings 15 days after emergence.

B Concentrations mg dm^−3^ B	Cultivar 1		Cultivar 2	
	Shoot	Root	Shoot	Root
0.5	5.390	27.157	5.612	27.157
30	14.615	16.020	16.722	15.930
60	16.364	18.525	8.072	13.939
120	30.563	20.421	23.069	17.155

B—boron. DW—dry weight.

## Data Availability

Data are contained within the article and Supplementary Materials.

## Supplementary Materials

The following supporting information can be downloaded at https://www.mdpi.com/article/10.3390/plants14040576/s1. Table S1: Analysis of variance for treatment effects using the significance of the F ratio (F-test) values for emergence velocity index, emergence percentage, shoot and root dry weight, and shoot and root length of seedlings of *Gossypium hirsutum* L.r. *latifolium* Hutch subjected to boron contamination. Treatments—B concentrations. Cultivars—TMG 50 WS3 and FM 911 GLTP. Table S2: Analysis of variance for treatment effects using the significance of the F ratio (F-test) values for amino acid concentrations in roots, stems, leaves, and cotyledons of *Gossypium hirsutum* L.r. *latifolium* Hutch seedlings subjected to boron contamination. Treatments—B concentrations. Cultivars—TMG 50 WS3 and FM 911 GLTP. Table S3: Analysis of variance for treatment effects using the significance of the F ratio (F-test) values for total soluble protein concentrations in roots, stems, leaves, and cotyledons of *Gossypium hirsutum* L.r. *latifolium* Hutch seedlings subjected to boron contamination. Treatments—B concentrations. Cultivars—TMG 50 WS3 and FM 911 GLTP. Table S4: Analysis of variance for treatment effects using the significance of the F ratio (F-test) values for phenolic compound concentrations in roots, stems, leaves, and cotyledons of *Gossypium hirsutum* L.r. *latifolium* Hutch seedlings subjected to boron contamination. Treatments—B concentrations. Cultivars—TMG 50 WS3 and FM 911 GLTP. Figure S1: (A) Root length for both cultivars, throughout B treatments; (B) root length for each cultivar separately, regardless of treatment; (C) shoot dry weight for both cultivars, throughout B treatments; (D) shoot dry weight of each cultivar regardless of treatment; (E) root dry weight for both cultivars, throughout B treatments; and (F) root dry weight of each *G. hirsutum* cultivar regardless of treatment. Means represented by different letters differ from each other by the Tukey test at 5% probability (*p* ≤ 0.05). Plot without regression equation represents data that did not fit the linear or quadratic curve.
