# Supplementary material for: Effects of Boron on the Emergence and Allocation of Metabolic Compounds in Two Herbaceous Cotton Cultivars
Source: Plants (Basel). 2025 Feb 13;14(4):576. doi: 10.3390/plants14040576 (PMC11858813; doi:10.3390/plants14040576)
Supplement: Supplementary file 1 [file plants-14-00576-s001.zip › plants-3439306-supplementary.pdf]

Plants

Effects of Boron on the emergence and allocation of metabolic compounds in two herbaceous cotton cultivars

Roberta Possas de Souza, Maycon Anderson de Araujo, Lucas Baltazar Longhi, Isabella Fiorini de Carvalho, Bruno Bonadio Cozin and Liliane Santos de Camargos *

Department of Biology and Zootechny, School of Engineering, São Paulo State University (UNESP), Ilha Solteira 15385-000, Brazil; roberta.possas@unesp.br (R.P.d.S.); maycon.araujo@unesp.br (M.A.d.A.); lucas.longhi@unesp.br (L.B.L.); if.carvalho@unesp.br (I.F.d.C.); bruno.bonadio-cozin@unesp.br (B.B.C.)

* Correspondence: liliane.camargos@unesp.br; Tel.: +55-18-37431152

Table S1. Analysis of variance for treatment effects using the significance of the F ratio (F-test) values for emergence velocity index, emergence percentage, shoot and root dry weight, shoot and root length of seedlings of *Gossypium hirsutum* L.r. *latifolium* Hutch subjected to boron contamination. Treatments – B concentrations. Cultivars – TMG 50 WS3 and FM 911 GLTP.

SV	EVI	Emergence (%)	S-DW	R-DW	S-L	R-L
Treatments (A)	32.844**	16.5425**	12.8233**	1.66162^{ns}	62.693**	8.0039**
Cultivars (B)	31.774**	9.4036**	0.9484^{ns}	0.97961^{ns}	21.128**	23.8364**
A x B	3.613*	4.4776*	0.7345^{ns}	0.82796^{ns}	3.542*	1.4296^{ns}
CV%	15.62	14.12	18.6	80.41	8.83	19.65

SV – source of variation, CV – coefficient of variation, EVI – emergence velocity index, S-DW – shoot dry weight. R-DW – root dry weight, S-L – shoot length, R-L - root length. **Significant at 1% ($p < 0.01$); * Significant at 5% ($p < 0.05$); ns - not significant.

Table S2. Analysis of variance for treatment effects using the significance of the F ratio (F-test) values for amino acids concentrations in roots, stems, leaves and cotyledons of *Gossypium hirsutum* L.r. *latifolium* Hutch seedlings subjected to boron contamination. Treatments – B concentrations. Cultivars – TMG 50 WS3 and FM 911 GLTP.

SV	Roots	Stems	Leaves	Cotyledons
Treatments (A)	14.6756**	6.956**	1.896^{ns}	49.497**
Cultivars (B)	23.5894**	252.208**	185.609**	1.196^{ns}
A x B	3.8923*	13.990**	10.652**	25.304**
CV%	19.27	11.94	8.13	12.06

SV – source of variation, CV – coefficient of variation. **Significant at 1% ($p < 0.01$); * Significant at 5% ($p < 0.05$); ns - not significant.

Table S3. Analysis of variance for treatment effects using the significance of the F ratio (F-test) values for total soluble proteins concentrations in roots, stems, leaves and cotyledons of *Gossypium hirsutum* L.r.

latifolium Hutch seedlings subjected to boron contamination. Treatments – B concentrations. Cultivars – TMG 50 WS3 and FM 911 GLTP.

SV	Roots	Stems	Leaves	Cotyledons
Treatments (A)	6.6176**	18.454**	193.82**	292.163**
Cultivars (B)	21.3214**	80.960**	377.96**	229.310**
A x B	9.8401**	6.164**	35.05**	64.327**
CV%	27.84	15.17	9.49	7.24

SV – source of variation, CV – coefficient of variation. **Significant at 1% (p < 0.01).

Table S4. Analysis of variance for treatment effects using the significance of the F ratio (F-test) values for phenolic compounds concentrations in roots, stems, leaves and cotyledons of *Gossypium hirsutum* L.r. *latifolium* Hutch seedlings subjected to boron contamination. Treatments – B concentrations. Cultivars – TMG 50 WS3 and FM 911 GLTP.

SV	Roots	Stems	Leaves	Cotyledons
Treatments (A)	1.0493^{ns}	7.2410**	57.430**	259.798**
Cultivars (B)	18.2097**	6.3409*	28.697**	5.131*
A x B	5.5178**	3.9521*	8.820**	42.331**
CV%	26.68	24.45	15.33	12.84

SV – source of variation, CV – coefficient of variation. **Significant at 1% (p < 0.01); * Significant at 5% (p < 0.05); ns - not significant.

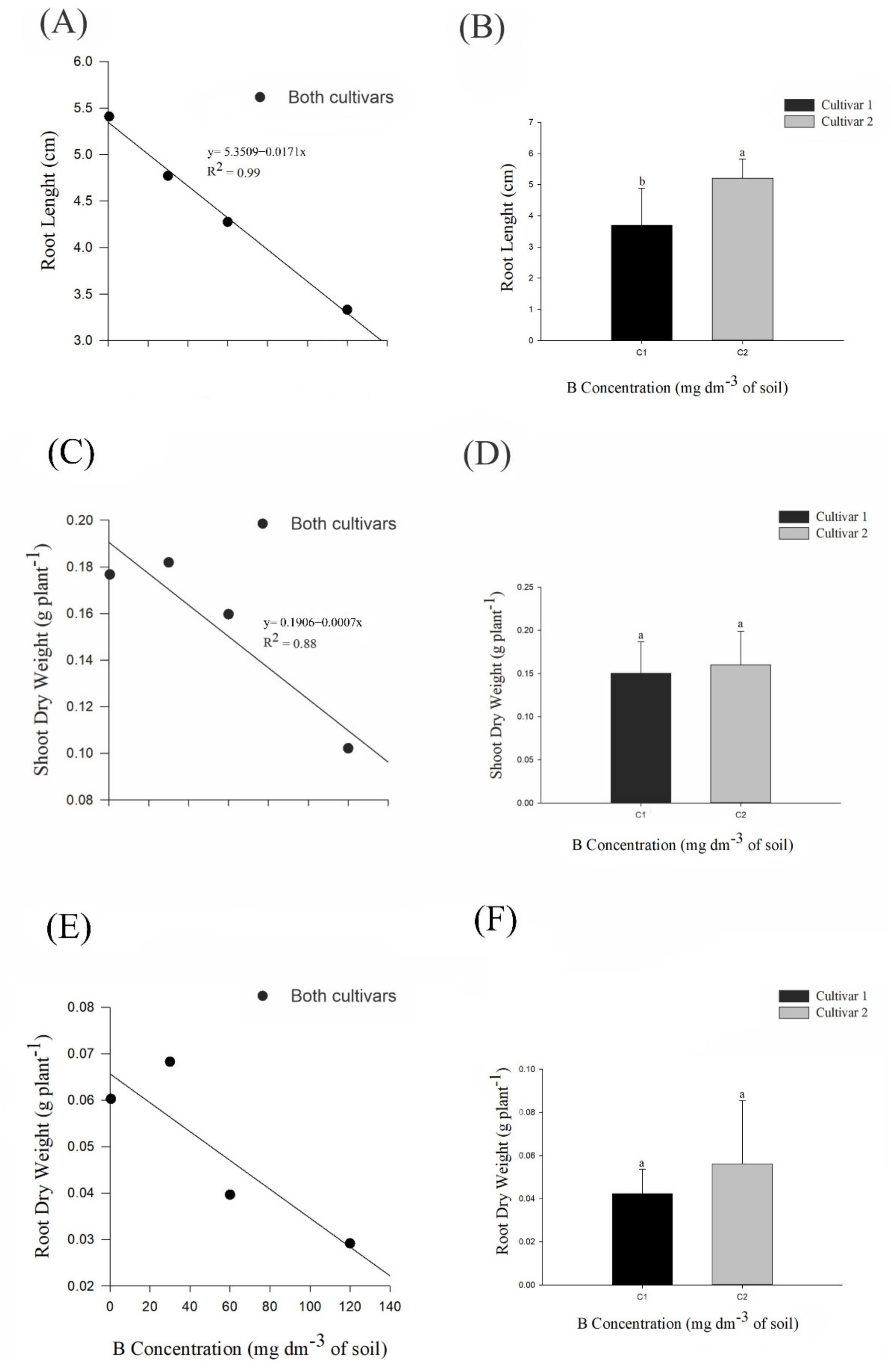

Figure S1: (A) Root Length for both cultivars, throughout B treatments; (B) Root length for each cultivar separately, regardless treatment; (C) shoot dry weight for both cultivars, throughout B treatments; (D) shoot dry weight of each cultivar regardless of treatment; (E) root dry weight for both cultivars, throughout B treatments, and (F) root dry weight of each *G. hirsutum* cultivar regardless of treatment. Means represented by different letters differ from each other by the Tukey test at 5% probability ($p \leq 0.05$). A plot without regression equation represents data that did not fit the linear or quadratic curve.
